# Smoking and Body Fat Mass in Relation to Bone Mineral Density and Hip Fracture: The Hordaland Health Study

**DOI:** 10.1371/journal.pone.0092882

**Published:** 2014-03-25

**Authors:** Jannike Øyen, Clara Gram Gjesdal, Ottar Kjell Nygård, Stein Atle Lie, Haakon E. Meyer, Ellen Margrete Apalset, Per Magne Ueland, Eva Ringdal Pedersen, Øivind Midttun, Stein Emil Vollset, Grethe S. Tell

**Affiliations:** 1 Department of Rheumatology, Haukeland University Hospital, Bergen, Norway; 2 Department of Global Public Health and Primary Care, University of Bergen, Bergen, Norway; 3 Department of Clinical Science, University of Bergen, Bergen, Norway; 4 Department of Heart Disease, Haukeland University Hospital, Bergen, Norway; 5 Department of Clinical Dentistry, University of Bergen, Bergen, Norway; 6 Section for Preventive Medicine and Epidemiology, University of Oslo, Oslo, Norway; 7 Norwegian Institute of Public Health, Division of Epidemiology, Oslo, Norway; 8 Laboratory of Clinical Biochemistry, Haukeland University Hospital, Bergen, Norway; 9 Bevital AS, Bergen, Norway; Johns Hopkins Bloomberg School of Public Health, United States of America

## Abstract

Lower bone mineral density (BMD) in smokers may be attributable to lower body weight or fat mass, rather than to a direct effect of smoking. We analyzed the effects of smoking exposure, assessed by plasma cotinine, and body fat on BMD and the risk of subsequent hip fracture. In the community-based Hordaland Health Study (HUSK), 3003 participants 46–49 years and 2091 subjects 71–74 years were included. Cotinine was measured in plasma and information on health behaviors was obtained from self-administered questionnaires. BMD and total body soft tissue composition were measured by dual X-ray absorptiometry. Information on hip fracture was obtained from computerized records containing discharge diagnoses for hospitalizations between baseline examinations 1997–2000 through December 31^st^, 2009. In the whole cohort, moderate and heavy smokers had stronger positive associations between fat mass and BMD compared to never smokers (differences in regression coefficient (95% CI) per % change in fat mass = 1.38 (0.24, 2.52) and 1.29 (0.17, 2.4), respectively). In moderate and heavy smokers there was a nonlinear association between BMD and fat mass with a stronger positive association at low compared to high levels of fat mass (Davies segmented test, p<0.001). In elderly women and men, heavy smokers had an increased risk of hip fracture compared to never smokers (hazard ratio = 3.31, 95% CI: 2.05, 5.35; p<0.001). In heavy smokers there was a tendency of a lower risk of hip fracture with higher percentage of fat mass. The deleterious effect of smoking on bone health is stronger in lean smokers than in smokers with high fat mass.

## Introduction

Cigarette smoking is associated with low bone mineral density (BMD) [1], [2] and increased risk of fracture [1]–[4] in both women and men. The mechanisms behind the negative effects of smoking on bone are not completely known, but it is partly related to the influence from smoking on sex hormones in both genders [5]. A direct adverse effect of smoking on skeletal remodeling and bone cells has also been suggested [6], and in an animal model impaired bone formation was seen during nicotine exposure [7]. Further, smoking may decrease calcium absorption [8] and parathyroid hormone concentration [9].

Low body weight and low body mass index (BMI) are associated with low BMD [10], but the association between low BMI and fracture risk seems to be site specific. In some previous studies low BMI was associated with hip [11]–[13] and lower extremities fractures [12], while in a meta-analysis low BMI was associated with increased risk of all types of fractures [14]. In recently published studies the results are conflicting [15], [16]. Low fat mass has been associated with increased risk of vertebral fractures in men [17] and hip fractures in women [11]. Accordingly, fat mass has been found to be positively associated with BMD in both women and men in some [18]–[20], but not all [21], [22] studies. In a recently published meta-analysis including 44 studies, only four reported negative associations between fat mass and BMD in any participant subgroups [23]. Biochemical interactions between adipose tissue and bone are complex and linked by a multitude of pathways involving cytokines, adiponectin, insulin, and leptin, and hormones like insulin and estrogen [24].

On average, smokers have lower weight and lower fat mass than non-smokers [25]. In a Danish study on perimenopausal women a significant interaction was found between low fat mass (<13.3 kg) and current smoking on femoral neck BMD, demonstrating that among women with high fat mass (>19.0 kg) smoking did not affect BMD [25]. A Norwegian study described a 3-fold increased risk of hip fracture in lean (BMI≤20 kg/m^2^) female smokers compared to lean non-smokers, but no increased risk in smokers with BMI above the population mean (>25 kg/m^2^) [3]. In men a 2-fold increased risk of hip fracture was found in smokers compared to non-smokers independently of BMI [3].

Studies of the association between smoking, BMD and fracture risk have mostly been based on self-reported smoking [1], [2] with its inherent limitations. Cotinine is the main metabolite of nicotine and a sensitive marker of recent active and passive tobacco exposure. While nicotine has a half-life of only 2–3 hours, cotinine has a half-life of 11–37 hours [26].

To our knowledge, plasma cotinine-based classification of smoking habits has not been studied as a predictor of risk of hip fractures, and the effect of smoking exposure and fat mass on BMD and hip fracture risk has not been reported. We hypothesized that there is a higher risk of low BMD and hip fracture in lean smokers than in smokers with high fat mass. We also wanted to investigate the interaction between fat mass and smoking in relation to BMD and hip fracture.

The independent impact of fat mass and lean mass on BMD has been previously studied in this population [20]. The aim of the present study was to explore whether the deleterious effect of smoking on BMD and hip fracture risk was the same regardless of body composition/the proportion of fat mass.

## Methods

### Ethics statements

The study was approved by the Regional Committee for Medical Research Ethics review, REC West. Each participant signed an informed consent form.

### Study population

Men and women, residing in Bergen (Norway) and three neighboring municipalities, born 1925–1927 and 1950–1951 were invited for the main Hordaland Health Study (HUSK) where the baseline examination was done during 1998–2000. Those invited had participated in a previous study (The Hordaland Homocysteine Study) during 1992–93 [27]. Altogether 77% met for the main study and they received an invitation for densitometry measurements. A total of 5408 (73.8%) met for this substudy. Inclusion criteria for the present study were participants with valid total body and femoral BMD scans and plasma cotinine measurements. Thirty subjects were excluded because of invalid BMD scans or bilateral hip prostheses. Total body soft tissue composition was invalid in 183 subjects (motion or metal artifacts). Thirty-nine persons were excluded because of non-white ethnicity. Lastly, plasma cotinine measurements were missing in 62 subjects. Thus, 5094 participants (1821 women and 1182 men aged 46–49 years and 1126 women and 965 men aged 71–74 years) comprise the cohort of the current study. Follow-up time was from inclusion until first hip fracture, while the observations were censored at death or on December 31^st^, 2009. As only eight participants in the youngest age group were diagnosed with a hip fracture during the follow-up period, only the oldest age group was included in the hip fracture analyses. During the follow-up period, 218 women and 309 men died without having suffered a hip fracture.

### Bone mineral density and body composition

BMD and total body soft tissue composition was measured by dual X-ray absorptiometry (DXA) on a stationary fan beam densitometry (Expert-XL; Lunar Company Inc, Madison, Wis) by four trained technicians. The left hip was scanned except when there was a history of hip prosthesis or fracture. The results were based on measurements of femoral neck BMD as this measurement site is recommended as the reference standard for description of osteoporosis [28]. Fat mass (%) was calculated as fat mass (kg) in percent of total body weight (kg). Daily scanning of the manufacturer-supplied spine phantom showed no instrumentation drift and a coefficient of variation of <0.9% during the whole study period.

### Hip fractures

Information on hip fracture was attained from computerized records containing discharge diagnoses for all hospitalizations occurring between HUSK baseline examinations through December 31^st^, 2009 at the six hospitals in Hordaland County. Mean follow-up time was 9.8 years. Hip fracture was defined as the first fracture of the proximal femur occurring during the observation period. Only hip fractures confirmed by a concurrent code of an adequate surgical procedure were included in order to validate the fracture registration; all hospital discharges with an identified hip fracture diagnosis were searched for adequate surgical treatment. Further description of the classification codes is previously described [29]. Information on time of death was attained from the Norwegian Population Register.

### Other measurements

Height and weight were measured with light clothing. BMI was calculated as weight in kilograms divided by the square of height in meters. Self-administered questionnaires provided information on smoking, physical activity and hormone replacement therapy, which were missing in 13, 160 and 743 participants, respectively. Smoking habits were categorized as current, former or never-smokers. The number of cigarettes smoked daily was also collected. Leisure time physical activity was categorized as no or light regular activity (<1 hour/week), regular (1–2 hours/week) and hard regular activity (≥3 hours/week). Use of estrogen supplements was categorized as current or no use. Information on dietary intake was collected using a validated 169-item food frequency questionnaire (FFQ). FFQ was missing in 482 subject, and eight participants with energy intake extremes (<2.5 or >97.5 percentiles) were excluded.

### Determination of cotinine and categorization of smoking

Non-fasting blood samples were collected and plasma was stored at −80 degrees Celsius. Plasma cotinine concentration was measured by liquid chromatography-tandem mass spectrometry at the laboratory Bevital A/S (Bergen, Norway) [30]. Never smoking was defined as plasma cotinine levels less than 85 nmol/L [31] and no report of previous smoking. Former smoking was defined as self-reported previous smoking and plasma cotinine levels less than 85 nmol/L. Moderate smoking was defined as plasma cotinine levels between 85 and 1199 nmol/L and heavy smoking as 1200 nmol/L or higher. A plasma cotinine level of 1200 nmol/L corresponded to a self-reported consumption of about 15 cigarettes per day among our study participants.

### Statistical analyses

Categorical variables are expressed as numbers and percentages and continuous variables as means with standard deviations (SD). The hip fracture incidence rate per 10,000 person-years was calculated by summarizing the follow-up time (years) for the study population (person-years), and dividing the numbers of hip fractures on person-years. Linear representation of the categorical variables was used to test for trend. General linear regression models (GLM) with femoral neck BMD (g/cm^2^) as the dependent variable and fat mass (%) as independent variable were used for each category of smoking habits. GLM was used to investigate a possible interaction between fat mass (%) or BMI and smoking on BMD. BMD was expressed in terms of g/cm^2^ to give the direct strength of the relation between BMD and fat mass or BMI in each smoking category. To get more comprehensible regression coefficients, fat mass and BMI were divided by 1000 before the analyses. To test for nonlinearity in the slopes for the association between fat mass (%) and BMD for each of the different smoking categories, we used the Davies segmented test [32]. To graphically express the dose-response associations between fat mass (%) and BMD according to smoking categories, we used a generalized additive logistic regression model (GAM) with a smoothing spline term with adjustment for sex and age group.

Cox proportional hazards regression models were used to estimate associations between smoking categories in combination with fat mass (%) or BMI and subsequent hip fracture in the oldest subjects. These analyses were conducted with and without adjustments for physical activity and BMD. To study the dose-response associations between fat mass (%) and the risk of hip fracture according to smoking categories, a Cox model using a spline covariate smoothing with adjustment for sex was used. Two-tailed p-values <0.05 were considered statistically significant. The analyses were performed using SPSS for Windows (IBM SPSS Statistics 19). The generalized additive models were computed with the R (http://cran.r-project.org) package “gam” [33] and the Cox spline smoothed curves were constructed using the R package “survival”.

## Results

### Study population

Characteristics of the female and male participants are shown in Tables 1 and 2, respectively. In women of both age groups, BMI (kg/m^2^), fat mass (%), fat mass (kg), lean mass (kg), and BMD (g/cm^2^) were significantly lower in heavy smokers than in moderate-, former-, and never smokers (Table 1). A similar trend was found among the oldest men (Table 2). In the youngest men, lean mass and BMD were significantly lower in heavy smokers compared with the other smoking categories. Among the youngest women and men, heavy smokers were less physically active than moderate-, former-, and never smokers. No significant differences between the smoking groups were found for total energy intake and use of estrogen supplementation in elderly women. During follow-up, 100 women and 52 men in the oldest age group suffered a hip fracture. The hip fracture incidence rates per 10,000 person-years were 83.5 for women and 54.2 for men. Among the oldest women, a higher proportion of heavy smokers suffered a hip fracture compared to moderate and never smokers.

**Table 1:** Characteristics of the female study participants by baseline age group and smoking categories.

	*Women 46–49 years old*						*Women 71–74 years old*					
	All		Smoking categories^b^			P for	All		Smoking categories^b^			P for
	subjects^c^	Never	Former	Moderate	Heavy	trend^d^	subjects^c^	Never	Former	Moderate	Heavy	trend^d^
Number of participants	1821	728	423	341	329		1126	677	275	108	66	
	(100)	(40.0)	(23.2)	(18.7)	(18.1)		(100)	(60.1)	(24.4)	(9.6)	(5.9)	
Body mass index (kg/m^2^)	24.8	25.2	25.1	24.9	23.6	<0.001	26.2	26.3	27.1	25.0	23.0	<0.001
	(4.0)	(4.3)	(3.9)	(3.9)	(3.5)		(4.2)	(3.9)	(4.6)	(4.3)	(3.4)	
Total fat mass (kg)	24.5	25.4	25.1	24.7	21.5	<0.001	27.0	27.3	29.1	24.0	20.2	<0.001
	(9.7)	(10.1)	(9.7)	(9.8)	(8.2)		(9.6)	(9.1)	(10.4)	(9.3)	(8.0)	
Total lean mass (kg)	40.3	40.5	40.5	40.5	39.6	0.006	37.6	37.8	38.0	37.0	35.1	<0.001
	(4.5)	(4.6)	(4.2)	(4.9)	(4.4)		(4.3)	(4.0)	(4.4)	(4.7)	(4.5)	
Fat mass (%)	36.6	37.3	37.1	36.8	34.2	<0.001	40.6	40.9	42.2	38.0	35.3	<0.001
	(7.8)	(7.8)	(7.6)	(7.6)	(7.8)		(8.2)	(7.7)	(8.1)	(9.1)	(8.7)	
Fat mass <15%	6	1	0	0	5		8	2	1	3	2	
	(0.3)	(0.1)			(1.5)		(0.7)	(0.3)	(0.4)	(2.8)	(3.0)	
Fat mass >40%	577	263	138	108	68	<0.001	635	396	173	46	20	<0.001
	(31.7)	(36.1)	(32.6)	(31.7)	(20.7)		(56.4)	(58.5)	(62.9)	(42.6)	(30.3)	
Total energy (kJ/day)	7947.0	7827.8	8042.5	8140.2	7879.3	0.381	6575.9	6561.3	6573.4	6625.2	6666.5	0.701
	(2340.0)	(2193.7)	(2191.4)	(2642.8)	(2489.6)		(2221.1)	(2183.5)	(2188.9)	(2427.0)	(2470.3)	
No regular physical activity	655	241	140	116	158	<0.001	443	273	94	51	25	0.987
	(36.4)	(33.6)	(33.3)	(34.6)	(48.2)		(43.1)	(44.5)	(36.4)	(51.5)	(43.1)	
Estrogen supplementation	342	96	99	77	70	<0.001	125	72	29	15	9	0.406
	(25.1)	(17.9)	(30.6)	(30.9)	(27.5)		(14.9)	(14.6)	(13.6)	(17.6)	(18.8)	
Femoral neck BMD (g/cm^2^)	0.96	0.98	0.96	0.95	0.93	<0.001	0.76	0.77	0.77	0.74	0.73	0.002
	(0.12)	(0.13)	(0.12)	(0.12)	(0.13)		(0.11)	(0.11)	(0.12)	(0.12)	(0.13)	
Hip fracture during follow-up^e^	4	1	2	-	1	0.858	100	51	22	9	18	<0.001
	(0.2)	(0.1)	(0.5)		(0.3)		(8.9)	(7.5)	(8.0)	(8.3)	(27.3)	

The Hordaland Health Study^a^.

Abbreviations: BMD, bone mineral density; BMI, body mass index.

a Values are given as mean (standard deviation (SD)) for continuous variables and number (%) for categorical variables.

b Never smoking, plasma cotinine levels <85 nmol/L and no self-reported previous smoking; former smoking, self-reported previous smoking and plasma cotinine levels <85 nmol/L; moderate smoking, plasma cotinine levels between 85 and 1199 nmol/L; heavy smoking, plasma cotinine levels ≥1200 nmol/L.

c Number of participants with data on femoral neck BMD, body soft tissue composition and plasma cotinine.

d *P*-value for trend across smoking categories.

e Hip fractures from inclusion in 1997–99 until December 31^st^, 2009.

Total numbers may vary between variables due to varying numbers of missing data.

**Table 2:** Characteristics of the male study participants by baseline age group and smoking categories.

	*Men 46–49 years old*						*Men 71–74 years old*					
	All		Smoking categories^b^			P for	All		Smoking categories^b^			P for
	subjects^c^	Never	Former	Moderate	Heavy	trend^d^	subjects^c^	Never	Former	Moderate	Heavy	trend^d^
Number of participants	1182	404	349	188	241		965	245	555	87	78	
	(100)	(34.2)	(29.5)	(15.9)	(20.4)		(100)	(25.4)	(57.5)	(9.0)	(8.1)	
Body mass index (kg/m^2^)	26.2	25.9	26.8	26.4	25.6	0.379	26.0	25.8	26.3	25.7	24.3	0.002
	(3.3)	(3.2)	(3.4)	(3.3)	(3.3)		(3.1)	(3.0)	(3.0)	(3.5)	(3.3)	
Total fat mass (kg)	20.6	19.6	22.5	20.8	19.4	0.690	21.2	19.9	22.4	20.4	17.8	0.202
	(9.0)	(8.4)	(9.7)	(8.4)	(8.9)		(8.5)	(7.9)	(8.5)	(8.6)	(7.9)	
Total lean mass (kg)	59.9	60.0	60.9	60.2	58.4	0.003	55.0	55.4	55.3	53.9	53.6	0.005
	(6.2)	(6.1)	(6.2)	(6.2)	(6.0)		(5.8)	(5.7)	(5.8)	(5.5)	(6.0)	
Fat mass (%)	24.7	23.9	26.1	24.9	23.9	0.981	27.0	25.7	28.1	26.6	23.9	0.315
	(7.4)	(7.2)	(7.3)	(7.1)	(7.8)		(7.4)	(7.3)	(7.2)	(7.1)	(7.7)	
Fat mass <15%	107	43	19	14	31		55	19	20	5	11	
	(9.1)	(10.6)	(5.4)	(7.4)	(12.9)		(5.7)	(7.8)	(3.6)	(5.7)	(14.1)	
Fat mass >40%	31	9	14	3	5	0.360	34	7	24	3	0	0.119
	(2.6)	(2.2)	(4.0)	(1.6)	(2.1)		(3.5)	(2.9)	(4.3)	(3.4)		
Total energy (kJ/day)	10547.4	10415.7	10509.4	10409.1	10953.5	0.073	8605.6	8775.5	8510.1	8531.2	8858.3	0.878
	(2870.6)	(2632.0)	(2799.1)	(2985.2)	(3252.8)		(2471.1)	(2467.6)	(2502.8)	(2528.9)	(2157.0)	
No regular physical activity	445	131	126	75	113	<0.001	273	58	156	40	19	0.059
	(38.0)	(32.7)	(36.3)	(40.3)	(47.7)		(29.3)	(24.3)	(29.2)	(47.6)	(25.3)	
Femoral neck BMD (g/cm^2^)	0.99	1.00	1.00	0.97	0.95	<0.001	0.90	0.93	0.90	0.86	0.86	<0.001
	(0.13)	(0.13)	(0.14)	(0.13)	(0.12)		(0.14)	(0.15)	(0.14)	(0.12)	(0.14)	
Hip fracture during follow-up^e^	4	2	2	-	-	0.231	52	10	29	6	7	0.079
	(0.3)	(0.5)	(0.6)				(5.4)	(4.1)	(5.2)	(6.9)	(9.0)	

The Hordaland Health Study^a^

Abbreviations: BMD, bone mineral density; BMI, body mass index.

a Values are given as mean (standard deviation (SD)) for continuous variables and number (%) for categorical variables.

b Never smoking, plasma cotinine levels <85 nmol/L and no self-reported previous smoking; former smoking, self-reported previous smoking and plasma cotinine levels <85 nmol/L; moderate smoking, plasma cotinine levels between 85 and 1199 nmol/L; heavy smoking, plasma cotinine levels ≥1200 nmol/L.

c Number of participants with data on femoral neck BMD, body soft tissue composition and plasma cotinine.

d *P*-value for trend across smoking categories.

e Hip fractures from inclusion in 1997–99 until December 31^st^, 2009.

Total numbers may vary between variables due to varying numbers of missing data.

### Smoking, fat mass and bone mineral density

For all participants, BMD and fat mass were positively associated in all smoking categories, with the strongest association among moderate and heavy smokers (Table 3). Significant differences in the association (regression coefficients) between fat mass and BMD between the different smoking categories were found. Both heavy and moderate smokers had significantly stronger associations between fat mass and BMD compared to never smokers (p = 0.018 and p = 0.024, respectively) after adjustment for sex and age group (Table 3). The explained variance was 0.287 (28.7%) and an overall test of the model gave a significant fit (F(9,5073) = 226.5, p<0.001). After additional adjustment for physical activity, the association was significant among heavy smokers only (p = 0.034) (Table 3). The same tendency was observed after stratifying on sex and age groups, although the results were borderline significant (e.g. the oldest heavy smoking women: differences in regression coefficient = 4.64 per % change in fat mass, 95% confidence interval (CI): −0.07, 9.29; p = 0.050).

**Table 3:** Linear associations between fat mass (%) and femoral neck BMD (g/cm^2^) for each category of smoking for all participants (n = 5094) in the Hordaland Health Study.

	Adjusted for sex and age						Adjusted for sex, age and physical activity					
	Regression coefficients			Differences in regression coefficients			Regression coefficients			Differences in regression coefficients		
Smoking categories^a^	B	95% CI	P value	B	95% CI	P value	B	95% CI	P value	B	95% CI	P value
Heavy	3.43	2.40, 4.47	<0.001	1.38	0.24, 2.52	0.018	3.60	2.54, 4.65	<0.001	1.26	0.10, 2.41	0.034
Moderate	3.35	2.33, 4.36	<0.001	1.29	0.17, 2.41	0.024	3.32	2.28, 4.36	<0.001	0.98	−0.17, 2.12	0.093
Former	1.30	0.59, 2.01	<0.001	-0.75	−1.60, 0.10	0.082	1.58	0.86, 2.31	<0.001	−0.76	−1.62, 0.10	0.084
Never	2.05	1.44, 2.67	<0.001	0 (ref.)	(-,-)	-	2.34	1.71, 2.98	<0.001	0 (ref.)	(-,-)	-

General linear regression models showing the regression coefficient between fat mass and BMD for each smoking category, and the differences in regression coefficients per % change in fat mass for each category, with never smokers as the reference group.

Abbreviations: BMD, bone mineral density (g/cm^2^); B, Beta; CI, confidence interval.

a Never smoking, plasma cotinine levels less than 85 nmol/L and no self-reported previous smoking; former smoking, self-reported previous smoking and plasma cotinine level >85 nmol/L; moderate smoking, plasma cotinine levels between 85 and 1199 nmol/L; heavy smoking, plasma cotinine levels ≥1200 nmol/L.

Davies segmented test showed that for heavy and moderate smokers there was a nonlinear association, with a steeper positive association between BMD and fat mass at low compared to high values of fat mass, and a significant breakpoint at fat mass of 18.4% in heavy smokers, and at 21.1% in moderate smokers (p<0.001).

**Figure 1:**
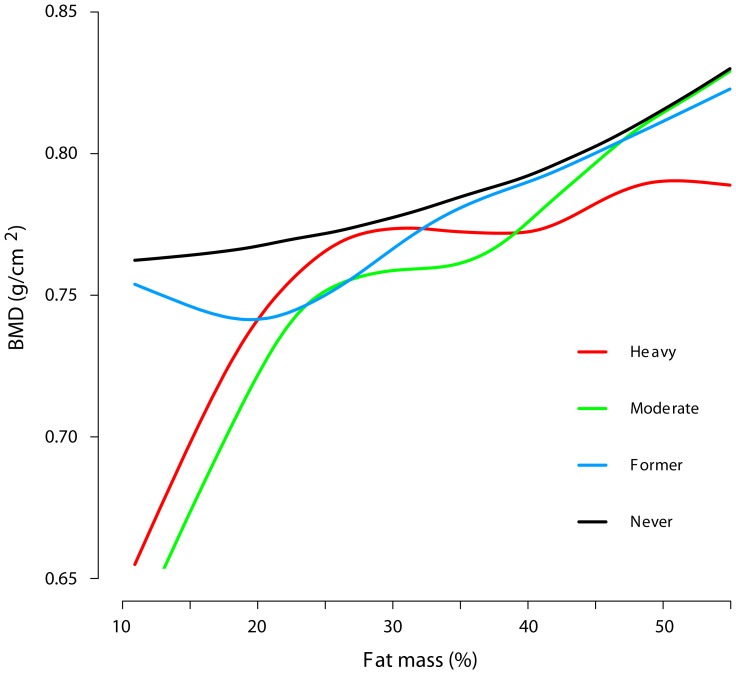
Dose-response curves between fat mass (%) and femoral neck bone mineral density (BMD, g/cm^2^) for different smoking categories by generalized additive model (GAM). All participants (n = 5094) are included in the models, which are adjusted for sex and age group (The Hordaland Health Study). Never smoking is defined as plasma cotinine levels <85 nmol/L and no self-reported previous smoking; former smoking, self-reported previous smoking and plasma cotinine levels <85 nmol/L; moderate smoking, plasma cotinine levels between 85 and 1199 nmol/L; heavy smoking, plasma cotinine levels ≥1200 nmol/L.

The non-linear dose-response associations (functional form) between BMD and fat mass are shown in figure 1. The curves show that BMD increased with increasing levels of fat mass, with the steepest increases among heavy and moderate smokers with low fat mass.

The associations were somewhat stronger for BMI as compared to fat mass (Table S1, Table 3).

### Smoking, fat mass and hip fracture

For elderly women and men combined, heavy smokers had an increased risk of hip fracture compared to never smokers (hazard ratio = 3.31, 95% CI: 2.05, 5.35; p<0.001) after adjustment for sex and fat mass. Fat mass was not significantly related to risk of hip fracture in any of the smoking categories after adjustment for sex. However, there was a tendency towards heavy smokers having the strongest fracture risk reduction with increasing fat mass (Table 4). There were no significant differences between the associations for the different smoking categories (Table 4). Adjustment for physical activity and BMD did not materially change the results (Table 4). Similar results were found when BMI was used in the analyses instead of fat mass (Table S2).

**Table 4:** Associations between fat mass (%) and risk of hip fracture according to smoking status in elderly women and men (n = 2091) in the Hordaland Health Study.

	Adjusted for sex						Adjusted for sex, physical activity and BMD					
	HR for hip fracture by fat mass			Differences in HR			HR for hip fracture by fat mass			Differences in HR		
Smoking categories^a^	HR	95% CI	P value	HR	95% CI	P value	HR	95% CI	P value	HR	95% CI	P value
Heavy	0.67	0.42, 1.07	0.091	0.78	0.44, 1.37	0.391	0.80	0.62, 1.03	0.082	0.87	0.61, 1,24	0.424
Moderate	0.79	0.47, 1.32	0.368	0.92	0.50, 1.69	0.791	0.87	0,69, 1.11	0.259	0.95	0.68, 1.31	0.738
Former	1.05	0.73, 1.50	0.798	1.23	0.77, 1.96	0.394	0.92	0.65, 1.30	0.643	0.95	0.62, 1.45	0.811
Never	0.85	0.62, 1.19	0.348	1.00	(-,-)	-	0.88	0.62, 1,24	0.452	1.00	(-,-)	-

Cox proportional hazards regression models showing hazard ratio between fat mass and hip fracture within each smoking category, and differences in HRs between each smoking category compared to never smokers.

Abbreviations: HR, hazard ratio; CI, confidence interval.

a Never smoking, plasma cotinine levels <85 nmol/L and no self-reported previous smoking; former smoking, previous self-reported smoking and plasma cotinine levels <85 nmol/L; moderate smoking, plasma cotinine levels between 85 and 1199 nmol/L; heavy smoking, plasma cotinine levels ≥1200 nmol/L.

**Figure 2:**
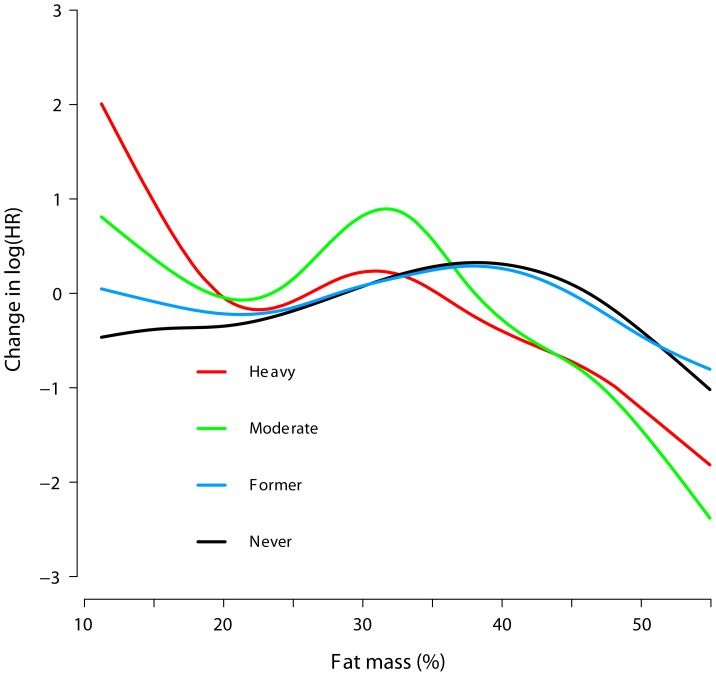
Dose-response curves between fat mass (%) and risk of hip fracture according to smoking status in elderly women and men (n = 2091) by generalized additive Cox modeling. The curves are adjusted for sex (The Hordaland Health Study). Never smoking is defined as plasma cotinine levels <85 nmol/L and no self-reported previous smoking; former smoking, previous self-reported smoking and plasma cotinine levels <85 nmol/L; moderate smoking, plasma cotinine levels between 85 and 1199 nmol/L; heavy smoking, plasma cotinine levels ≥1200 nmol/L.

The association (functional form) between fat mass and the risk of hip fracture is shown in figure 2, and reveals a non-linear dose-response relation. There was a tendency towards decreasing risk of hip fracture with increasing levels of fat mass for each of the smoking categories, with the steepest decrease among heavy smokers with low fat mass.

## Discussion

In this study the positive association between fat mass and BMD was stronger among moderate and heavy smokers compared to never smokers, and the relation was stronger in smokers with low compared to high fat mass or BMI. In elderly women and men, an increased risk of hip fracture was associated with heavy smoking. Fat mass was not significantly related to risk of hip fracture in any of the smoking categories, but the tendency was that heavy smokers had higher risk of fractures at lower levels of fat mass, and that this risk decreased markedly with increasing fat mass.

Strengths of this study are the large number of participants of both genders, the population based cohort design, and the long follow-up time of more than 10 years for hip fractures. However, smoking status, BMD and fat mass were measured only at baseline; we therefore have no information on potential changes in these or in other factors during follow-up. All BMD and body composition measurements were performed on the same DXA machine. We measured cotinine, which have been reported to correlate better than self-reports with various effects of smoking [34]. A total number of 185 participants who reported no current smoking had plasma cotinine levels above 85 nmol/L. Misclassification might be related to under-reporting and/or passive smoking. Data on smokeless tobacco use was not collected, thus some of the participants with high cotinine levels might have been snuffers and not cigarette smokers. On the other hand, during 1998–2000, snuffing was very rare among adults and elderly in Norway, and we do not consider this to be of importance in the interpretation of our findings. In support of the latter, our results did not materially change when we used self-reported current smoking status instead of plasma cotinine in the analyses. Alcohol abuse is a potential confounding factor, but here were no major differences in reported alcohol intake within the different smoking categories, and adjustment for alcohol intake did not alter the results (data not shown). In addition, diabetes could also be a confounding factor since persons with diabetes usually have higher fat mass [35]. However, 166 HUSK participants reported diabetes and/or use of anti-diabetic medication, and 100 of these had fat mass above 30% (51 above 40%). Exclusion of these participants or including diabetes as a covariate did not alter the results.

Percent fat mass was positively related to BMD in all smoking categories in our study. In the meta-analysis by Ho-Pam et al [23], studies in which negative correlations between fat mass and BMD were found included few subjects (n<100) and the negative correlation coefficients reported were overall weaker than the (positive) correlations found in other studies. The significant association between fat mass and BMD may reflect a role of nutrition and sex hormones in bone building and bone tissue maintenance [23]. We found a stronger relation between fat mass and BMD in smokers than non-smokers and in smokers with low compared to high fat mass. This suggests that among smokers, the relative impact of fat tissue as an endocrine organ may be more important among those with little fat mass.

It has been suggested that lower BMD in smokers may be attributable to lower body weight rather than to a direct effect of smoking [36]. However, our results do not support that lower BMI is the predominant explanation as both moderate and heavy smokers with low fat mass had lower BMD compared to never smokers with low fat mass. This is in accordance with a meta-analysis demonstrating a negative effect of smoking on BMD independent of differences in weight between smokers and non-smokers [2]. On the other hand, we found that smokers with low fat mass had significantly lower BMD than smokers with high fat mass. Thus, the negative effect of smoking on BMD seems to be especially deleterious among lean people.

To our knowledge, there are no published studies using cotinine to investigate the interaction with fat mass on BMD and subsequent hip fracture. However, urinary cotinine was investigated in relation to BMD in a study of pre- and postmenopausal Korean females, and a significant dose-related effect of smoking on BMD was observed [37]. In addition, serum cotinine in relation to bone mineral content (BMC) was investigated in a large cohort study in the USA and high serum cotinine levels were found to be a significant risk factor for low BMC in both women and men [38]. However, the interaction between smoking and fat mass was not examined in these studies. We found lower BMD in both moderate and heavy smokers with low fat mass compared to never smokers with low fat mass. Our results are similar with findings from a Danish study on 2015 perimenopausal women where a significant interaction was found between self-reported current smoking and fat mass in the lowest tertile (<13.3 kg) on femoral neck BMD, but not among those in the highest tertile of fat mass (>19.0 kg) [25]. Two studies demonstrated a higher risk of low BMD in current smokers compared with non-smokers in both genders, after adjustment for BMI [1], [39]. In addition, a dose-dependent effect of cigarette smoking (cigarettes/day, years smoked) on BMD has been demonstrated in both genders; larger exposure was associated with lower BMD [2], [40].

We observed an increased risk of hip fracture in elderly heavy smokers compared to never smokers. The association did not change materially after adjustment for physical activity and BMD, which suggests that increased fracture risk among heavy smokers may be independent of BMD. However, we did not find any significant interaction between fat mass or BMI and the different smoking categories on the risk of hip fracture which may be due to the relative small number of hip fractures. In a large Norwegian study a relative risk of 1.5 (95% CI: 1.0, 2.4) for suffering a hip fracture was found in female self-reported smokers with BMI below the population mean (25 kg/m^2^), compared to lean non-smokers, increasing to a 3-fold RR when cut-off for BMI was set at 20 kg/m^2^ [3]. On the other hand, a population-based case-control study from Sweden showed an increased risk of hip fracture in female smokers compared to never smokers (odds ratio = 1.66, 95% CI: 1.41, 1.95), but with similar results after dividing the group according to BMI above and below the median [41]. Hemenway et al [42], did not find any significant association between cigarettes smoked per day and risk of hip fracture among women, nor any interaction with body weight. Forsen et al [3], found an increased risk of hip fractures in male smokers independently of BMI. In some other studies on male smokers the results are conflicting regarding impact of BMI or weight on risk of fracture [43], [44].

We did not find any significant differences in the association between fat mass and the risk of low BMD or hip fracture in former smokers compared to never smokers. In some previous studies with adjustment for BMI, smoking cessation was found to both decrease the risk of low BMD [2], [40] and hip fracture [44] as well as to increase the risk of hip fracture [45]. The number of years smoked and time since quitting may be important [2]. In the study by Hollenback et al [40], previous smokers with short time since smoking cessation had lower hip BMD than never smokers and long-term quitters, while current smokers had the lowest BMD. In a large Norwegian study of women and men under 75 years, the risk of hip fracture was higher in former smokers, even among those who had quit smoking more than 5 years before, compared to never smokers [45].

The mechanisms behind the negative effects of smoking on bone are not completely known. In women, positive associations between tobacco smoking, premature menopause [25], low estrogen levels [5] and low BMD have been found. In addition, a more rapid bone loss after menopause in smokers than non-smokers has been observed [46]. It has been estimated that smoking increases the lifetime risk of sustaining a hip fracture by 31% in women and 40% in men [2], and bone loss has been reported to be more rapid among male than female smokers [47]. The apparently higher risk among men may be due to a higher total consumption of cigarettes among male smokers, or a protective effect of hormone replacement therapy among women [2]. However, in the current study the prevalence of hip fracture was higher among female than male smokers. Smoking has also been shown to reduce testosterone levels in men [48]. A adverse effect of smoking on skeletal remodeling and bone cells has been observed [6]. Further, smoking may affect other hormones, minerals and enzymes involved in bone regulation resulting in reduced calcium absorption [8] and parathyroid hormone concentration [9]. Increased oxidative stress and inflammation are also observed in smokers [49].

Generally, and as we observed, smokers have lower BMI and lower fat mass than non-smokers [25]. It has been suggested that smoking has an appetite-suppressing effect, thereby leading to lower energy intake. However, we and others [50] did not find any significant differences in the total energy intake between smokers and non-smokers. A modestly increased rate of energy metabolism in smokers compared to non-smokers due to release of hormones as e.g. adrenaline from the central nervous system has been suggested [51].

The biochemical pathways linking adipose tissue to bone are complex [24]. For example, in women a decreased rate of bone loss might be explained by the production of extragonadal estrogen in adipose tissue after menopause. Thus, postmenopausal women with low fat mass have lower estrogen production and therefore a higher risk of osteoporosis [24]. In elderly men, androgen deficiency caused by hypogonadism may contribute to bone loss, but androgen deficiency is not related to fat mass [52]. However, low estradiol levels in elderly men may also cause faster bone loss [16].

Our findings indicate that the positive relation between fat mass and BMD was stronger among moderate and heavy smokers compared to never smokers, and the deleterious effect was stronger in smokers with low compared to high fat mass. Thus, physicians should be particularly attentive to lean smokers concerning the risk of osteoporosis. An uplifting finding in our study as well as in other studies [2] is that it seems that smoking cessation may slow down or partially reverse the accelerated bone loss caused by years of smoking. There is no convincing evidence that fat mass *per se* protects against osteoporosis or hip fractures. For a deeper understanding, further research should aim at investigating through which pathways fat tissue may exert a protective effect on bone exposed to toxic substances.

## Supporting Information

Table S1 Linear associations between BMI (kg/cm^2^) and femoral neck BMD (g/cm^2^) for each category of smoking for all participants (n = 5094) in the Hordaland Health Study. General linear regression models showing the regression coefficient between BMI and BMD for each smoking category, and the differences in regression coefficients per unit change in BMI for each category, with never smokers as the reference group.
(DOCX)

Table S2 Associations between BMI (kg/cm^2^) and risk of hip fracture according to smoking status in elderly women and men (n = 2091) in the Hordaland Health Study. Cox proportional hazards regression models showing hazard ratio between BMI and hip fracture within each smoking category, and differences in HRs between each smoking category compared to never smokers.
(DOCX)
