# Correction: Smoking and Body Fat Mass in Relation to Bone Mineral Density and Hip Fracture: The Hordaland Health Study

**DOI:** 10.1371/journal.pone.0101335

**Published:** 2014-06-24

**Authors:** The PLOS ONE Staff

The dates provided for baseline examinations are incorrect in the Abstract and Tables 1 and 2. The correct 5^th^ sentence of the Abstract is:

“Information on hip fracture was obtained from computerized records containing discharge diagnoses for hospitalizations between baseline examinations 1998–2000 through December 31^st^, 2009.”

The authors have provided a correct version of Table 1 and Table 2 below.

**Table 1:** Characteristics of the female study participants by baseline age group and smoking categories.

	*Women 46-49 years old*						*Women 71-74 years old*					
	All subjects^c^	Smoking categories^b^				P for trend^d^	All subjects^c^	Smoking categories^b^				P for trend^d^
		Never	Former	Moderate	Heavy			Never	Former	Moderate	Heavy	
Number of participants	1821 (100)	728 (40.0)	423 (23.2)	341 (18.7)	329 (18.1)		1126 (100)	677 (60.1)	275 (24.4)	108 (9.6)	66 (5.9)	
Body mass index (kg/m^2^)	24.8 (4.0)	25.2 (4.3)	25.1 (3.9)	24.9 (3.9)	23.6 (3.5)	<0.001	26.2 (4.2)	26.3 (3.9)	27.1 (4.6)	25.0 (4.3)	23.0 (3.4)	<0.001
Total fat mass (kg)	24.5 (9.7)	25.4 (10.1)	25.1 (9.7)	24.7 (9.8)	21.5 (8.2)	<0.001	27.0 (9.6)	27.3 (9.1)	29.1 (10.4)	24.0 (9.3)	20.2 (8.0)	<0.001
Total lean mass (kg)	40.3 (4.5)	40.5 (4.6)	40.5 (4.2)	40.5 (4.9)	39.6 (4.4)	0.006	37.6 (4.3)	37.8 (4.0)	38.0 (4.4)	37.0 (4.7)	35.1 (4.5)	<0.001
Fat mass (%)	36.6 (7.8)	37.3 (7.8)	37.1 (7.6)	36.8 (7.6)	34.2 (7.8)	<0.001	40.6 (8.2)	40.9 (7.7)	42.2 (8.1)	38.0 (9.1)	35.3 (8.7)	<0.001
Fat mass <15%	6 (0.3)	1 (0.1)	0	0	5 (1.5)		8 (0.7)	2 (0.3)	1 (0.4)	3 (2.8)	2 (3.0)	
Fat mass >40%	577 (31.7)	263 (36.1)	138 (32.6)	108 (31.7)	68 (20.7)	<0.001	635 (56.4)	396 (58.5)	173 (62.9)	46 (42.6)	20 (30.3)	<0.001
Total energy (kJ/day)	7947.0 (2340.0)	7827.8 (2193.7)	8042.5 (2191.4)	8140.2 (2642.8)	7879.3 (2489.6)	0.381	6575.9 (2221.1)	6561.3 (2183.5)	6573.4 (2188.9)	6625.2 (2427.0)	6666.5 (2470.3)	0.701
No regular physical activity	655 (36.4)	241 (33.6)	140 (33.3)	116 (34.6)	158 (48.2)	<0.001	443 (43.1)	273 (44.5)	94 (36.4)	51 (51.5)	25 (43.1)	0.987
Estrogen supplementation	342 (25.1)	96 (17.9)	99 (30.6)	77 (30.9)	70 (27.5)	<0.001	125 (14.9)	72 (14.6)	29 (13.6)	15 (17.6)	9 (18.8)	0.406
Femoral neck BMD (g/cm^2^)	0.96 (0.12)	0.98 (0.13)	0.96 (0.12)	0.95 (0.12)	0.93 (0.13)	<0.001	0.76 (0.11)	0.77 (0.11)	0.77 (0.12)	0.74 (0.12)	0.73 (0.13)	0.002
Hip fracture during follow-up^e^	4 (0.2)	1 (0.1)	2 (0.5)	-	1 (0.3)	0.858	100 (8.9)	51 (7.5)	22 (8.0)	9 (8.3)	18 (27.3)	<0.001

The Hordaland Health Study^a^

Abbreviations: BMD, bone mineral density; BMI, body mass index.

a Values are given as mean (standard deviation (SD)) for continuous variables and number (%) for categorical variables.

b Never smoking, plasma cotinine levels <85 nmol/L and no self-reported previous smoking; former smoking, self-reported previous smoking and plasma

cotinine levels <85 nmol/L; moderate smoking, plasma cotinine levels between 85 and 1199 nmol/L; heavy smoking, plasma cotinine levels ≥1200 nmol/L.

c Number of participants with data on femoral neck BMD, body soft tissue composition and plasma cotinine.

d *P*-value for trend across smoking categories.

e Hip fractures from inclusion in 1998-2000 until December 31^st^, 2009.

Total numbers may vary between variables due to varying numbers of missing data.

**Table 2:** Characteristics of the male study participants by baseline age group and smoking categories.

	*Men 46-49 years old*						*Men 71-74 years old*					
	All subjects^c^	Smoking categories^b^				P for trend^d^	All subjects^c^	Smoking categories^b^				P for trend^d^
		Never	Former	Moderate	Heavy			Never	Former	Moderate	Heavy	
Number of participants	1182 (100)	404 (34.2)	349 (29.5)	188 (15.9)	241 (20.4)		965 (100)	245 (25.4)	555 (57.5)	87 (9.0)	78 (8.1)	
Body mass index (kg/m^2^)	26.2 (3.3)	25.9 (3.2)	26.8 (3.4)	26.4 (3.3)	25.6 (3.3)	0.379	26.0 (3.1)	25.8 (3.0)	26.3 (3.0)	25.7 (3.5)	24.3 (3.3)	0.002
Total fat mass (kg)	20.6 (9.0)	19.6 (8.4)	22.5 (9.7)	20.8 (8.4)	19.4 (8.9)	0.690	21.2 (8.5)	19.9 (7.9)	22.4 (8.5)	20.4 (8.6)	17.8 (7.9)	0.202
Total lean mass (kg)	59.9 (6.2)	60.0 (6.1)	60.9 (6.2)	60.2 (6.2)	58.4 (6.0)	0.003	55.0 (5.8)	55.4 (5.7)	55.3 (5.8)	53.9 (5.5)	53.6 (6.0)	0.005
Fat mass (%)	24.7 (7.4)	23.9 (7.2)	26.1 (7.3)	24.9 (7.1)	23.9 (7.8)	0.981	27.0 (7.4)	25.7 (7.3)	28.1 (7.2)	26.6 (7.1)	23.9 (7.7)	0.315
Fat mass <15%	107 (9.1)	43 (10.6)	19 (5.4)	14 (7.4)	31 (12.9)		55 (5.7)	19 (7.8)	20 (3.6)	5 (5.7)	11 (14.1)	
Fat mass >40%	31 (2.6)	9 (2.2)	14 (4.0)	3 (1.6)	5 (2.1)	0.360	34 (3.5)	7 (2.9)	24 (4.3)	3 (3.4)	0	0.119
Total energy (kJ/day)	10547.4 (2870.6)	10415.7 (2632.0)	10509.4 (2799.1)	10409.1 (2985.2)	10953.5 (3252.8)	0.073	8605.6 (2471.1)	8775.5 (2467.6)	8510.1 (2502.8)	8531.2 (2528.9)	8858.3 (2157.0)	0.878
No regular physical activity	445 (38.0)	131 (32.7)	126 (36.3)	75 (40.3)	113 (47.7)	<0.001	273 (29.3)	58 (24.3)	156 (29.2)	40 (47.6)	19 (25.3)	0.059
Femoral neck BMD (g/cm^2^)	0.99 (0.13)	1.00 (0.13)	1.00 (0.14)	0.97 (0.13)	0.95 (0.12)	<0.001	0.90 (0.14)	0.93 (0.15)	0.90 (0.14)	0.86 (0.12)	0.86 (0.14)	<0.001
Hip fracture during follow-up^e^	4 (0.3)	2 (0.5)	2 (0.6)	-	-	0.231	52 (5.4)	10 (4.1)	29 (5.2)	6 (6.9)	7 (9.0)	0.079

The Hordaland Health Study^a^

Abbreviations: BMD, bone mineral density; BMI, body mass index.

a Values are given as mean (standard deviation (SD)) for continuous variables and number (%) for categorical variables.

b Never smoking, plasma cotinine levels <85 nmol/L and no self-reported previous smoking; former smoking, self-reported previous smoking and plasma

cotinine levels <85 nmol/L; moderate smoking, plasma cotinine levels between 85 and 1199 nmol/L; heavy smoking, plasma cotinine levels ≥1200 nmol/L.

c Number of participants with data on femoral neck BMD, body soft tissue composition and plasma cotinine.

d *P*-value for trend across smoking categories.

e Hip fractures from inclusion in 1998-2000 until December 31^st^, 2009.

Total numbers may vary between variables due to varying numbers of missing data.
