# Supplementary material for: Smoking and Body Fat Mass in Relation to Bone Mineral Density and Hip Fracture: The Hordaland Health Study
Source: PLoS One. 2014 Mar 25;9(3):e92882. doi: 10.1371/journal.pone.0092882 (PMC3965480; doi:10.1371/journal.pone.0092882)
Supplement: Table S1 — Linear associations between BMI (kg/cm2) and femoral neck BMD (g/cm2) for each category of smoking for all participants (n = 5094) in the Hordaland Health Study. General linear regression models showing the regression coefficient between BMI and BMD for each smoking category, and the differences in regression coefficients per unit change in BMI for each category, with never smokers as the reference group. (DOCX) [file pone.0092882.s001.docx]

**Supplemental Table S1** Linear associations between BMI (kg/cm^2^) and femoral neck BMD (g/cm^2^) for each category of smoking for all participants (n = 5094) in the Hordaland Health Study. General linear regression models showing the regression coefficient between BMI and BMD for each smoking category, and the differences in regression coefficients per unit change in BMI for each category, with never smokers
as the reference group.

|  | Adjusted for sex and age | | | | | | Adjusted for sex, age and physical activity | | | | | |
| --- | --- | --- | --- | --- | --- | --- | --- | --- | --- | --- | --- | --- |
|  | Regression coefficients | | | Differences in regression coefficients | | | Regression coefficients | | | Differences in regression coefficients | | |
| Smoking categories^a^ | B | 95% CI | P value | B | 95% CI | P value | B | 95% CI | P value | B | 95% CI | P value |
| Heavy | 13.51 | 10.43, 16.59 | <0.001 | 5.79 | 2.29, 9.29 | 0.001 | 13.87 | 10.77, 16.97 | <0.001 | 5.55 | 2.02, 9.08 | 0.002 |
| Moderate | 10.59 | 7.80, 13.39 | <0.001 | 2.87 | -0.38, 6.13 | 0.083 | 10.62 | 7.76, 13.49 | <0.001 | 2.30 | -1.02, 5.62 | 0.174 |
| Former | 7.79 | 5.83, 9.74 | <0.001 | 0.07 | -2.50, 2.63 | 0.961 | 8.88 | 6.89, 10.86 | <0.001 | 0.56 | -2.04, 3.15 | 0.674 |
| Never | 7.72 | 6.05, 9.39 | <0.001 | 0 (ref.) | (-,-) | - | 8.32 | 6.64, 10.01 | <0.001 | 0 (ref.) | (-,-) | - |

Abbreviations: BMI, body mass index; BMD, bone mineral density (g/cm^2^); B, Beta; CI, confidence interval.

^a^ Never smoking, plasma cotinine levels less than 85 nmol/L and no self-reported previous smoking; Former smoking, self-reported previous smoking and plasma cotinine level >85 nmol/L; Moderate smoking, plasma cotinine levels between 85 and 1199 nmol/L; Heavy smoking, plasma cotinine levels ≥1200 nmol/L.
