# Supplementary material for: Smoking and Body Fat Mass in Relation to Bone Mineral Density and Hip Fracture: The Hordaland Health Study
Source: PLoS One. 2014 Mar 25;9(3):e92882. doi: 10.1371/journal.pone.0092882 (PMC3965480; doi:10.1371/journal.pone.0092882)
Supplement: Table S2 — Associations between BMI (kg/cm2) and risk of hip fracture according to smoking status in elderly women and men (n = 2091) in the Hordaland Health Study. Cox proportional hazards regression models showing hazard ratio between BMI and hip fracture within each smoking category, and differences in HRs between each smoking category compared to never smokers. (DOCX) [file pone.0092882.s002.docx]

**Supplemental Table S2** Associations between BMI (kg/cm^2^) and risk of hip fracture according to smoking status in elderly women and men
(n = 2091) in the Hordaland Health Study. Cox proportional hazards regression models showing hazard ratio between BMI and hip fracture within each smoking category, and differences in HRs between each smoking category compared to never smokers.

|  | Adjusted for sex | | | | | | Adjusted for sex, physical activity and BMD | | | | | |
| --- | --- | --- | --- | --- | --- | --- | --- | --- | --- | --- | --- | --- |
|  | HR for hip fracture by BMI | | | Differences in HR | | | HR for hip fracture by BMI | | | Differences in HR | | |
| Smoking categories^a^ | HR | 95% CI | P value | HR | 95% CI | P value | HR | 95% CI | P value | HR | 95% CI | P value |
| Heavy | 0.79 | 0.60, 1.03 | 0.077 | 0.86 | 0.60, 1.24 | 0.426 | 0.94 | 0.72, 1.22 | 0.619 | 0.98 | 0.68, 1.41 | 0.905 |
| Moderate | 0.80 | 0.62, 1.04 | 0.090 | 0.88 | 0.63, 1.23 | 0.465 | 0.91 | 0.71, 1.17 | 0.463 | 0.95 | 0.68, 1.33 | 0.771 |
| Former | 0.84 | 0.58, 1.21 | 0.348 | 0.92 | 0.60, 1.42 | 0.713 | 0.93 | 0.66, 1.33 | 0.705 | 0.98 | 0.64, 1.50 | 0.913 |
| Never | 0.91 | 0.64, 1.30 | 0.607 | 1.00 | (-,-) | - | 0.96 | 0.67, 1.37 | 0.809 | 1.00 | (-,-) | - |

Abbreviations: BMI, body mass index; HR, hazard ratio; CI, confidence interval.

^a^ Never smoking, plasma cotinine levels <85 nmol/L and no self-reported previous smoking; former smoking, previous self-reported smoking and plasma cotinine levels
<85 nmol/L; moderate smoking, plasma cotinine levels between 85 and 1199 nmol/L; heavy smoking, plasma cotinine levels ≥1200 nmol/L.
